# Advancing Professional Education in Clinical Sexology: An Introduction to the Special Issue on Professional Education in Clinical Sexology

**DOI:** 10.1080/19317611.2026.2661795

**Published:** 2026-07-03

**Authors:** Elsa Almås, Leiszle Lapping-Carr

**Affiliations:** a Specialist in clinical psychology and clinical sexology (NACS), Ordbruk as, University of Agder, Kristiansand, Norway; b Department of Psychiatry & Behavioral Sciences, Northwestern University Feinberg School of Medicine, Chicago, IL, USA

**Keywords:** Sexual health, sexology, clinical sexology, professional education, global survey

## Abstract

This Special Issue of the International Journal of Sexual Health presents results from a survey on professional education in clinical sexology conducted by the WAS Committee for Professional Education in Clinical Sexology (WAS PES). WAS PES was first established in 2018 as a sub-committee of the Education committee, and in 2022 became a separate, ad hoc committee for professional education in clinical sexology. The committee includes members from all six of the regional federations of WAS. The committee chose to focus on clinical education and training, because this sub-field of sexology was identified as having the greatest gap between professional recommendations and training realities. The results of this survey confirm this view of professional education in clinical sexology. The survey demonstrated that there is much activity in this field, especially in Europe, North America, and South America. There are also strong programs in Australia, while programs are lacking in other parts of Asia/Oceania, the Eastern Mediterranean countries, and in both North and Sub-Saharan Africa. In this special issue, separate articles present the global results of the survey, and the results from each of the six WAS federations: Asia-Oceania Federation for Sexology (AOFS), Eastern Mediterranian Federation for Sexual Health (EMFeSH), European Federation of Sexology (EFS), Latin American Federation of Sexology and Sex Education Societies (FLASSES), North American Federation of Sexuality Organizations (NAFSO), and Sexual Health Africa (SHA). In addition to reporting on the results of the WAS PES survey, the authors of each regional report engaged in an epistemological study of the field of clinical sexology in their region. As a result, these authors highlight strengths and areas for improvement in their regions, noting the lack of governmental acknowledgement of sexology as particularly challenging. Still, a primary takeaway identified across regions is the positive and important role of clinical sexological training in enhancing WAS’s mission to advance sexual health and well-being for everyone.

## Introduction

### Importance of sexual health

Sexual problems affect individuals globally, and The World Health Organization (WHO) has integrated sexual health into public health concerns since 1975 (WHO, 1975). In 2015, sexual health was included as target 3.7 in the UN Sustainable Development Goals (SDG). In 2019, the International Classification of Diseases, 11^th^ Revision (ICD-11, WHO’s diagnostic manual) revised and depathologized many sexual health concerns, such as sexual dysfunctions and gender incongruence, moving from the chapters about mental and somatic problems to a new chapter on Sexual Health (Reed et al., 2016).

Sexual problems include a wide range of health, intrapersonal, and interpersonal concerns. Sexual problems can be bodily dysfunctions, relational, or result from disease, medical treatment, or social restrictions or injunctions. Many problems develop through individual conflict with cultural or societal expectations, differences in economic and personal privileges, and differences in power and autonomy. Sexual problems can be associated with alcohol and drug abuse (Edwards, 1983), genital mutilation (Berggren et al., 2006), problematic sexual behavior (Braun-Harvey & Vigorito, 2016; Neves, 2021), minority stress (Meyer, 2015), sexual abuse and violence (Buttner, 2018), and sexual trauma (Almås & Benestad, 2021).

Given the breadth of presentation of sexual problems, it is not surprising to find high prevalence rates. A large study from the USA reported that 43% of women and 31% of men experience sexual problems, (Laumann et al., 1999). Another USA-based study reported the prevalence of erectile dysfunction as 24.2%, increasing to 52.2% for men over 75 years (Mark et al., 2024). An epidemiological study of female sexual dysfunction finds that 40% of women endorse some form of sexual inadequacy, although only 12-25% also endorse associated personal distress (Palacios et al., 2009). In a Norwegian survey from 2017, 27% of men and 19% of women reported sexual problems for which they needed help. The most prevalent problems for men and women were erectile problems and pain with intercourse, respectively (Dalen, 2018). Taken together, sexual dysfunctions and other sexual problems cause significant distress and impact the health and well-being of individuals around the world.

While it is important to acknowledge the variety and prevalence of sexual problems around the world, this deficit-focused approach limits the capacity for optimal sexuality. Striving for sexual health, as individuals and as a society, has been adopted as an alternative approach to working to remove or fix sexual problems. Sexual health has been defined multiple times across the years, and definitions tend to reflect the societies and historical epochs within which they are written. The WHO most recently defined sexual health as “*…a state of physical, emotional, mental and social well-being in relation to sexuality; it is not merely the absence of disease, dysfunction or infirmity. Sexual health requires a positive and respectful approach to sexuality and sexual relationships, as well as the possibility of having pleasurable and safe sexual experiences, free of coercion, discrimination and violence. For sexual health to be attained and maintained, the sexual rights of all persons must be respected, protected and fulfilled.” (WHO, 2006)*

A good definition of sexual health must allow for diverse experiences and presentations to be considered healthy (Haeberle & Gindorf, 1993; Rohleder & Flowers, 2018; Sandfort & Ehrhardt, 2004). It must center the advancement of positive and consensual sexual experiences, rather than simply the removal of negative or harmful sexual experiences. This conceptualization of sexual health allows us to more clearly link the need for accessible sexual health treatment to overall health.

Sexual problems can cause or contribute to many other problems (e.g., depression, relational problems, and other health conditions), and good sexual health has been shown to improve overall health and wellbeing for many individuals (Gianotten, 2015; Gianotten et al., 2007; Graugaard, 2017; Vasconcelos et al., 2024; Whipple et al., 2007). Numerous health benefits are associated with healthy sexual expression, including longer life expectancy, lower frequency of fatal coronary events, reduced incidences of breast cancer in both men and women, bolstering of the immune system, better general physical well-being, fitness, better reproductive health, more regular menstrual cycles, less menstrual cramps, less prostate cancer, pain management, quality of life, less depression and suicide, less stress, better self-esteem, better intimacy, social health benefits, and increased spiritual insight (Whipple et al., 2007). Sexologists are the researchers, clinicians, health promoters, and educators that have the skills to address sexual health problems at individual and systemic levels.

### The profession of sexology

Sexology as a professional and scientific field developed alongside psychology and psychiatry during the late 19^th^ century in Europe. The field provided legitimacy to researching and treating sexual concerns and increased knowledge about how sexual concerns can manifest. In 1886 *Richard von Krafft-Ebing* published “Psychopathia sexualis”, a collection of case histories of sexual practices (von Krafft-Ebing, 1997). The first scientific journal devoted entirely to sexual questions: “Archivo delle Psicopatie Sessuali” was published by the Italian psychiatrist *Pasqual Penta* in 1896. These publications used a professsional and scientific frame to disseminate the knowledge gained by sexologists through clinical work and research. This was a significant shift from the previous approach of relegating sexual concerns to the domain of forensics (Foucault, 1973).

From there, sexology developed into a more robust profession. Early organizations devoted to sexology included the Gay Rights Organization founded by *Magnus Hirschfeld* in Berlin in 1897. In 1907, the Berlin physician, Iwan Bloch published his study “The Sexual Life of Our Time”, where he called for the establishment of sexology (*Sexualwissenshaft*) as a scientific enterprise in its own right, combining the methods and insights of both the natural and the cultural sciences. The Institute for Sexology was founded by Magnus Hirschfeld in Berlin in 1919. Freud contributed to early sexology with his *Three essays On Sexuality* (Freud, 1905/1977) which introduce human sexuality as an area of interest for psychiatry and psychology. In 1926, Albert Moll organized the first International Congress for Sex Research. This was the first international professional conference of sexologists. The very early sexological activities in Europe were ended by the Nazi regime in Germany in the 1930s, marked by the burning of the library of Hirschfeld′s Institute of Sex Research in Berlin in 1933 (Haeberle & Gindorf, 1993).

Professional interest for sexology continued in other areas of the world, dominated by North America. In the USA, Alfred Kinsey and his research group re-invigorated sexology, following its suppression in Europe. Kinsey and colleagues published the report of sexual behavior in American men in 1948 (Kinsey et al., 1948) and of American women in 1953 (Kinsey et al., 1953).

For many years, sexual behaviors considered outside the norm, such as homosexuality, were pathologized. This view was challenged by sexologists like Alfred Kinsey and Albert Ellis. Kinsey and Ellis shifted sexology’s focus on pathology, writing extensively on healthy variations in sexuality and partnering. Ellis is credited as one of the founders of the sexual revolution in the 1960′s and was one of the first sexologists in the US (Reiss, 2008; Reiss & Ellis, 2002). He was also one of the founders of the Society for the Scientific Study of Sexuality (SSSS) in 1957 (Bullough, 1994).

During the 1960′s gynecologist William Masters, together with Virginia Johnson started their studies on sexual response and sexual problems. They developed a therapeutic tool, still taught and practiced as “sensate focus” (Masters & Johnson, 1966, 1970). Helen Singer Kaplan elaborated on the approach in her influential book “*The new sex therapy*” (Kaplan, 1974). These early clinical interventions formed the basis of clinical sexology as it exists today.

As these sex-positive developments, treatment methods for sexual problems and increased inclusivity of sex research and sexuality theories became more widely adopted in the field and accessible to the public, skepticism and resistance from conservative groups grew. Conservative advocacy efforts have caused significant challenges in the advancement of sexology, especially in the form of limited funding for research in human sexuality (Bullough, 1994). One of the most effective forms of this conservative resistance is the continued governmental passivity with regard to sexology. In most parts of the world, governments do not acknowledge sexology as a profession, nor do they endorse the use of sexology as a tool for the development of better sexual lives for individuals, sexually healthy societies, sexual rights, sexual justice, and sexual pleasure (Gruskin et al., 2019).

For many years, there was a consistent increase in the acceptance of sexual and gender diversity by the public as well as governmental acknowledgement of the rights of sexual and gender diverse individuals. However, there has been a growing backlash to the acceptance and protections newly offered to sexual and gender diverse individuals in the past five to ten years. In particular, there is significant skepticism about the appropriateness of gender affirming medical care (e.g., puberty blockers) for trans youth, the influence of social media on gender identity development, and the appropriateness of trans women and girls participating in team sports. A highly influential publication feeding this skepticism linked adolescent trans identity to social contagion via social media victimization and coined the term Rapid Onset Gender Dysphoria (ROGD) to describe the phenomenon (Littman, 2018). However, the study has been highly criticized for methodological flaws, including a reliance on parent reports of their child’s identity development and social media use, without any other sources of information. The publication was later corrected to remove causal implications that were originally included in the conclusions. Other significant publications fueling the conservative backlash include the UK-based and biased Cass Report, purporting there is insufficient evidence for the safety of gender affirming medical care, and the Bell v. Tavistock decision by the UK High Court (later reverse in the Court of Appeals), finding that youth under 16 are not competent to consent to gender affirming medical care (de Vries et al., 2021; WPATH et al., 2020).

The backlash taken many forms, including the Vatican, trans excluding feminists (TERFs), Trump, Evangelical Christians, and other conservative alliances (Butler, 2024; Galvão et al., 2026). As a consequence of these publications and strong conservative advocacy efforts, many countries have banned gender affirming medical treatments, leaving trans and gender diverse people in despair.

Still, clinical offerings and research in sexology continue to grow. Today, there are multiple scientific journals exclusively devoted to sexology (e.g., Archives of Sexual Behavior, Journal of Sex Research, Journal of Sexual Medicine, Journal of Sex and Marital Therapy, International Journal of Sexual Health, International Journal of Transgender Health, and others).

There are also sexological organizations and societies, ranging from local (e.g., Society of Australian Sexologists, Danish Association for Clinical Sexology), to regional (e.g., Society for Sex Therapy and Research [in USA and Canada], Sexual Health Africa, European Federation for Sexology), to international (e.g., World Association for Sexual Health, International Society for Sexual Medicine, World Professional Association for Transgender Health) (Almås et al., 2022).

Despite these advances, the term “sexology” is understood and used differently depending on the region and context. Sometimes “sexology” is used as it has been thus far in this paper: referring to a formal profession with significant academic training. However, “sexology”can also refer to something quite the opposite: a field in which individuals interested in sexuality, with little (if any) formal training, offer advice or services to improve others’ sex lives. The availability of this diversity of support for sexual health concerns is not inherently problematic. However, using a single term to refer to this wide range of training and expertise can result in confusion or dismissal of the field by those crafting policy for advancing public health. It can also result in people who seek help for complex sexual problems receiving insufficient, potentially harmful care. Therefore, it is important to establish sexology as a scientifically-founded profession, akin to medicine and psychology. All three of these fields research and offer clinical treatment for different aspects of human health. Indeed, it is vital that sexology is more fully and formally integrated into the health care system, as today there are very few health systems that offer a clear path for individuals seeking support of sexual concerns. Medicine and psychology do not offer proper education for helping people with sexual problems, leaving a significant gap in treatment options and availability. Clinical sexologists are few and far between, as are the professional training opportunities for this specialization, as will be detailed in this special issue.

For the purposes of this paper and the special issue, a useful conceptualization of sexology is that proposed by German sexologists Erwin Haeberle and Rolf Gindorf in 1992: *“Sexology shall mean that field of science dealing systematically with human sexuality: asking questions and trying to answer them in certain logical and methodological (“scientific”) procedures. Consequently, a sexologist shall mean a person who deals professionally, scientifically and exclusively or predominantly with human sexuality.”*p.27 (Haeberle & Gindorf, 1993)

Just as sexology is not consistently used in accordance with the above definition, the term “sexual health” is sometimes used interchangably with sexology. One way to clarify the disctinction between sexology and sexual health is to frame sexology as a *tool*, while sexual health is a *goal* that includes sexual rights, sexual pleasure and sexual justice. WAS changed its name from *World Association for Sexology* to *World Association for Sexual Health* in 2005 to better align with an evolving and more expansive mission (Mark et al., 2024). *Sexual health* as a designation for practice has a more societal scope that concerns sexual health services, sexuality education, and sexual health promotion through educational, legal, political and organizational work. All the distinct aspects of sexology, and all the ways that different countries and regions around the world train professionals in sexology, are working toward improving sexual health.

*Clinical sexology* was constructively redefined through the integration of the bio-psycho-social model, first introduced by George Engel (Engel, 1977), and later integrated across medical and psychological fields. The biological, psychological, and social aspects of a sexual health concern can each independently be described and understood by different professional domains of sexology: Practice, research, and theory (Engel, 1980).

Based on a co-constructionist understanding of the bio-psycho-social model (Butler, 2024), all factors are reciprocally modified by the interaction between them. Butler describes how, for example, the subjective experience of gender is a result of interactions among genetic, psychological, and social factors. Similarly, sexuality is a strong biological drive, modified by individual experiences in a cultural context. While the bio-psycho-social model is not without criticisms, it is generally regarded as a useful model and an important tool for promoting interdisciplinary work with sexual problems (Berry & Berry, 2013; Nimbi et al., 2021).

Through research and clinical work, sexologists develop tools and theories for understanding human sexuality, supporting positive sexual development, preventing sexual problems, and treating sexual problems where they occur. *Clinical sexology* is the treatment of sexual concerns by health professionals with expertise in sexology. These can be physicians, psychologists, psychotherapists, family and couple therapists, physiotherapists, midwives, nurses, social workers, occupational therapists, and others who work directly with clients presenting with problems related to sexuality. Through conversations about sexuality, clinical sexologists help clients to develop knowledge and skills to overcome obstacles to sexual health, such as those based in taboos, shame, and secrecy. Clients are also given tools to change maladaptive sexual behavior patterns.

Despite the high prevalence of sexual problems, and the existence of evidence-based treatment for sexual health concerns, most health care personnel are not provided education or training to address sexual problems. Both clinicians and clients seeking care often feel uncomfortable just discussing sexual concerns in health care settings (Metz & Seifert, 1993). The avoidance resulting from this discomfort limits clinicians’ ability to assess for potential sexual problems and provide appropriate referrals. Improving the integration and accessibility of clinical sexology training is necessary in order to advance sexual health worldwide.

## Clinical sexology training

In 1975 WHO published a report based on an expert meeting on sexual health: “*Education and treatment in human sexuality: The training of health professionals. Report of a WHO meeting*, 1975”(WHO, 1975). The report recommended foundational education and training in sexual health for all health professionals. In spite of tremendous growth in our field, this has not yet resulted in sufficient sexological training of health professionals or allocation of treatment options for sexual health problems in the 50 years since its publication. The path to correcting this pervasive gap in healthcare is through professional education. Professional education in clinical sexology includes a range of levels of specialization. It can provide foundational training in basic sexology to health care providers. It can also provide more advanced clinical training for counselors, psychotherapists, nurses, physical therapists, medical doctors (and other health care professionals) interested in specializing in the treatment of sexual health concerns. There are many strong models for clinical sexology training, but the effective implementation of these models has been slow and there remains significant room for improvement.

In 2003, at the European Federation for Sexology (EFS) congress in Limasol, Woet Gianotten presented an overview of how clinical sexology had developed into different professional specialities; these are described in the following, with our comments:

- *Common (“dysfunction”) sexology*, addressing problems with erection and ejaculation, sexual desire, orgasm, pain. It is also reasonable to include issues related to traumatized sexuality, relational problems and communication difficulties.
- *Gender sexology*, addressing problems related to gender identity. The diagnostic term “gender incongruence” introduced in ICD-11 communicates that there is incongruence between gender assigned at birth and subjective identity. Medical intervention to attain a safe experience of belongingness in one′s body is desperately needed for many individuals who experience gender incongruence (Baumeister & Leary, 1995; Benestad, 2010). Gender has become more commonly conceptualized as falling on a spectrum, and many individuals who experience some kind of gender incongruence benefit from psychosocial counseling in the exploration of their gender identity. There is also a growing awareness that trans people can preserve their fertility and have children. (Fielding, 2021; Neal & Davies, 2000).
- *Orientation sexology*, addresses *sexual identities*, having to do with finding a partner with a preferred gender or other personal characteristics. For individual and couple therapy with lesbian, gay, bisexual and trans people, it is important that therapists are familiar with a diversity of sexual practices, traumas of retention (hiding who you are), shame, and minority stress (Almås & Benestad, 2021; Dominic Davies & Prunas 2026; Meyer, 2015; Neves & Davies, 2023a, 2023b).
- *Paraphilic (“direction”) sexology* addresses management of sexual arousal patterns and belongingness to sexual subcultures (Benestad et al., 2015). Clinical work must also address out of control (obsessive) sexual behavior (OCSB) (Braun-Harvey & Vigorito, 2016) and compulsive sexual behavior disorder (CSBD) (Gola & Potenza, 2018; Kraus et al., 2018; Neves, 2021)
- *Forensic sexology* addresses illegal sexual practices, sentencing, treatment and rehabilitation, and prevention (Grubin, 2015; Money, 1990).
- *Medical sexology* addresses sexual problems and function related to medical conditions, and medical treatment of sexual problems in general (Porst & Buvat, 2006; Porst & Reisman, 2012).
- *Rehabilitation sexology* addresses rehabilitation after disease, or loss of bodily or mental function (Porst et al., 2008).
- *Oncosexology* addresses sexual problems related to cancer (Gianotten, 2003; Plagens-Rotman et al., 2024).

These specialties include medical, psychological, and cultural/social components that the clinician must consider for effective treatment of sexual health concerns. Both generalists and specialists are needed to effectively address the range of sexual health concerns that present in the population.

The PLISSIT model, originally described by Annon in 1976, is a widely used model of sexology intervention (Annon & Robinson, 1976). The PLISSIT model has been identified as the preferred approach to determine whether a sexual concern can be treated by a generalist with basic interventions, or whether the concern requires more advanced clinical sexology expertise (Almås & Landmark, 2010; Landmark et al., 2013).

**Figure 1.**
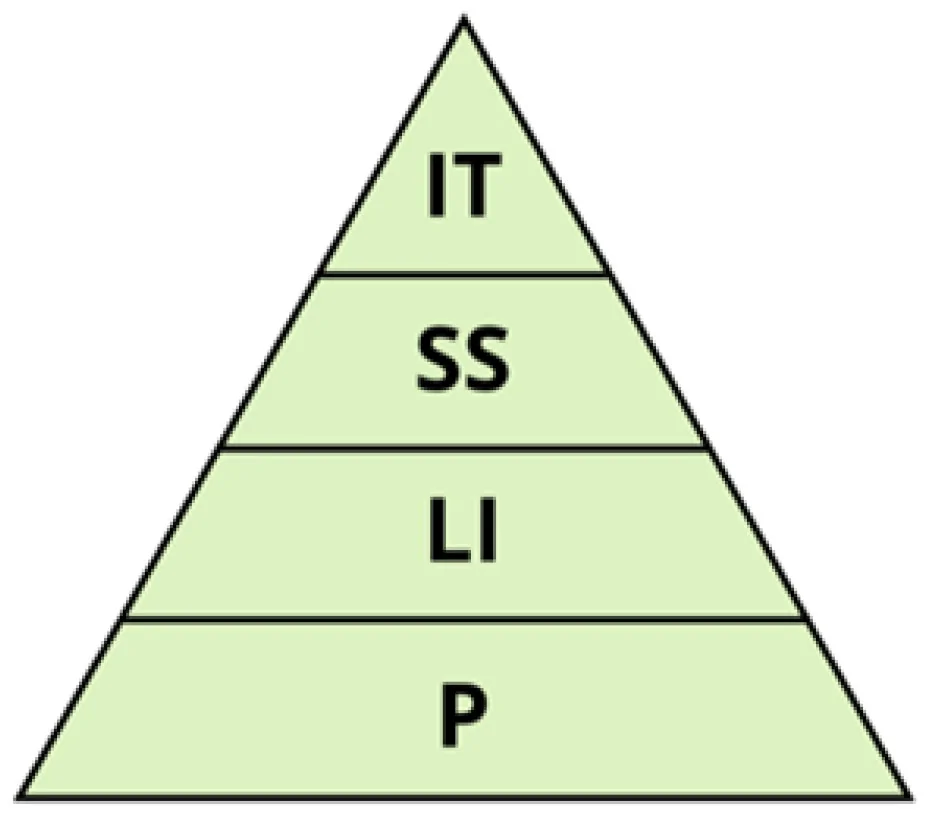
The PLISSIT model: Permission, limited information, specific suggestions, and intensive therapy.

The PLISSIT model illustrates both the presentation of problems and clinical approaches to address them and can be visualized as the pyramid seen in Figure 1:

*Permission* (P). The first level includes the largest number of problems, but also the largest group of people who can help. Permission to be sexual can be given by a family doctor, a general psychologist, a teacher, a school nurse, or a parent. Permission is the foundation of the model and is integral to all following levels of intervention.
*Limited Information* (LI). At this level, common concerns may include detailed information about the body, sexual feelings during puberty, why sexual feelings can be absent even if they are wanted, cultural norms, and legal regulations. The basic sexology knowledge needed for intervening at this level would ideally be integrated into the education of all health professionals.
*Specific Suggestions* (SS). The third level includes fewer problems and fewer professionals to address said problems. Most often, a specific suggestion is a targeted piece of advice about how to change a behavior to resolve a sexual concern. Health professionals will need some training in sexological counseling to intervene at this level. Most problems are solved on these three first levels: P, LI or SS.
*Intensive Therapy* (IT). The final level of the model requires specialtization in psychosexology or medical sexology for appropriate care. These are highly complex medical or psychological concerns that interact with sexual issues, such as having a neurological condition or experiencing sexual trauma.

In summary, the PLISSIT model demonstrates the benefits of interdisciplinary approaches for sexual health concerns, and emphasizes that most sexual problems can be solved without highly specialized expertise in clinical sexology. *Sexuality education* can prevent sexual problems from developing, and *clinical sexology* starts when problems have developed. Training in *basic sexology* will be sufficient for many problems and should be integrated into all health professional training. *Counseling sexology* (including P, LI and SS) can be given by most health professionals who receive additional training in sexology. *Intensive therapy* requires highly specialized psyschosexual or medical training. The gold-standard for care at the intensive sex therapy level is practiced in multidisciplinary centers, or at least with the possibility to consult specialists across disciplines, including gynecology, urology, endocrinology, psychology, trauma therapy, physiotherapy, neurology, and others as needed.

### Experiential training

An important part of training in clinical sexology is the Sexual Attitudes Reassessment (SAR), also called the Sexual Self Acknowledgement (SSA) in the Nordic countries. SAR is essential experiential training that challenges sexual values, attitudes, and beliefs and is the sensitivity training in the field of sexology. SAR consists of material and experiences that push the adult learner to confront values, attitudes, and beliefs about a wide range of issues within the broad field of human sexuality. SAR is a process of getting to know who you are – what you believe, feel, and think – and then enticing you to explore the vast world of human sexual expression (Britton & Dunlap, 2017). SSA has a similar intent, but has a slightly different focus; it is concerned with the sexologist as a professional and helping the trainee develop awareness of boundaries in oneself. Key questions participants reflect on include: What is ok for me to disclose about myself and my sexuality? To whom? How can I be aware of others’ boundaries? When I ask a question – what do I do with the information? How do I know when I’ve crossed the other person′s boundaries? Why should I respect boundaries – in myself - and in others? (Almås et al., 2019).

## The GLOPES^1^ survey

The work of the World Association for Sexual Health (WAS) is driven by its vision: sexual health and rights for all. WAS’s mission is as follows:

*“The World Association for Sexual Health (WAS) promotes and advocates for sexual health and sexual rights throughout the lifespan and across the world by advancing sexology, science-based sexuality knowledge, research, comprehensive sexuality education, clinical care and services. The purpose of WAS is to advance the international cooperation in the field of sexology by coordinating the activities designed to increase research and knowledge in sexology, including sexuality education, sexual health and the alleviation of sexual suffering, and the recognition and fulfillment of sexual rights throughout the world.”* (WAS′ statutes, §5).

In service of making progress toward this vision, it led an investigation into the current status of clinical sexology training. In line with WAS’s mission to advance sexual health globally, the WAS PES committee launched the GLOPES survey in 2023-2025, disseminating the survey through the WAS Newsletter to reach WAS members and affiliates. The GLOPES survey consisted of an electronic questionnaire administrated by the University of Agder. Detailed regional data were collected by members of the WAS PES-committee to obtain a more comprehensive understanding of unique considerations in different areas of the world. The long-term goal of the project is to develop educational guidelines and accreditation systems for education and training that can be applicable around the world.

The results of this global survey on professional education and training in clinical sexology are presented in this special issue.

The results show that there is a diversity of training programs in clinical sexology in all areas of the world. A global overview of the results, described by Lapping-Carr, Mastui-Santana, and Almås in this issue, shows that many trainings are not designed to fit into any form of accreditation system or to address specific levels of sexological education. The results show two primary subsets of training programs: Degree programs offered through universities, and continuing education programs based in sexological societies, NGOs and clinical settings. A large majority of respondents report a need for increased credibility for clinical sexology, while few report governmental recognition of sexology as a profession.

In the regional reports in this special issue, Africa: Smith, Nabwile Khisa, Obada Paul, Idowu, Ramlachan, and Rotich; Europe: Almås, Giami, and Kontula, North America: Lapping-Carr, Caffee, Gordon, and Medico; Latin America: Matsui-Santana, Valente, Brendler, and Urriola-González; East Meditteranean/North Africa: Assarag, El Fakahany, and El Kak; and Asia/Oceania: Lin and Lapping-Carr, the authors describe how cultural and contextual factors influence clinical sexology education and training. A commonality for many regions is the need to provide practical and necessary knowledge to professionals in whatever form possible to enable the treatment of sexual health concerns. Sometimes training and treatment are limited to basic sexology, and sometimes the full range from basic to highly specialized clinical sexology training and treatment is available. All points along this training and treatment spectrum are valuable, and it is necessary to be responsive to unique regional needs when developing clinical sexology training programs. The authors used the insights from their detailed regional analysis to identify strengths and significant areas for growth, and discuss how to leverage their strengths to meet the unique clinical sexology training needs of each region.

The project also identified different approaches to accreditation across regions. For example, the American Association for Sexuality Educators, Counselors and Therapists (AASECT), outlines a didactic curriculum and supervision requirements to obtain a certification as a sex counselor or sex therapist. These certifications require a license in a health care profession (e.g., clinical psychology, couple and family therapy, or medical doctor). The Nordic Association of Clinical Sexology (NACS) developed Nordic guidelines for authorization in sexology (Fugl-Meyer et al., 2001). These guidelines also require licensure in a health care profession before authorization as a sexologist. In Quebec, Canada, the Professional Order of Sexologists from Quebec was established in 2013, at which time sexology became a legally recognized and protected profession. In Quebec, there are degrees available at the bachelors, master’s, and doctoral levels. In the East Mediterranean region, clinical sexology training has been limited by traditional taboos, with few training opportunities available for health professionals. In Africa, training in clinical sexology is more typically approached in an ad hoc, informal fashion, with relatively few formal training programs for those with advanced degrees.

The GLOPES survey project was an ambitious effort and an impressive feat of international collaboration. Despite significantly different regional and local contexts, with different understandings of sexology and different approaches to training in sexology, the members of the WAS PES committee were able to work together effectively with collegiality. Through discussion, committee members were able to come to a consensus about how to approach such a large-scale survey, including how to ensure a methodological and data analytic approach and plan for dissemination that would effectively capture regional differences. The success of this project hinged on the active participation of the WAS PES committee members, who showed a remarkable commitment to and productivity in the completion of this project, and this resulting special issue.

## Key challenges

In most areas of the world, sexology lacks governmental recognition as a specialty, and information on sexual health is often truncated in health professional training programs.

The socio-political context worldwide is a major contributor to the lack of governmental recognition of clinical sexology. Even policy makers (like politicians and health system administrators) who want to improve sexual health, rights and justice for the public have not been able to follow the recommendations from WHO and WAS. Discomfort with discussing sexual health impedes the implementation of sexological education for professionals and the availability of treatment options. In some countries, it is still illegal to have sex with someone of the same gender or present as a gender different from that assigned at birth. In many cultures, openly discussing sexuality and sexual pleasure is taboo. The cultural stigma around sexuality can take many forms: Declarations of premarital sexuality as sinful, lack of sexuality education, fear of gender diversity, male power dominance and exploitation of women, silence around sexual violence and abuse, and shame around having been a victim of sexual abuse.

Despite several international declarations about sexual health, sexual rights, sexual pleasure, and sexual justice^2^, There has been little positive change at the national political or societal levels.

Even in countries with more sex positive cultures, there is little support for policies that advance sexual health. Sexuality is a difficult topic for many policymakers to openly discuss, as few have professional training in addressing sexual health. Policy makers may generally have a sex-positive stance, but they are also the products of an educational and healthcare system that does not adequately prepare people to deal with sexual concerns. They may be ignorant of the need, uncomfortable with discussing the topic, or not have the tools to change the guidelines for professional education. It is dependent on those with this training and comfort (i.e., sexologists) to advocate for policy change and demonstrate the necessity of formalizing and supporting education in clinical sexology.

Fortunately, there are strong advocates for inclusive and sex positive legislation and health policy. An effective approach to combating the conservative backlash will require understanding the fear behind their beliefs: People feel like they are “left behind” and experience inclusion as a threat toward safety and predictability. It will be necessary to address this fear and show that human rights for minoritized groups do not conflict with human rights for those with majority priviledge, be they Evangelical Christians or feminists. Sexual problems affect individuals from all ideological, political, and religious backgrounds.

## Conclusion

In this introduction to the special issue on professional education in clinical sexology, we have described sexology as a profession and its historical development. We have also discussed clinical sexology as a part of sexology and the broader sexual health field aimed at supporting people who experience sexual health concerns. We have described the PLISSIT-model as a training model for clinical sexology that could ideally be implemented across health professions to provide foundational training, with opportunities for advanced specialization in the field.

Advancing sexual health on a global scale requires developing comprehensive and contextually responsive professional education curricula. This developmental work starts with an accounting and description of the current state of professional education in clinical sexology. This special issue provides both broad quantitative descriptions of the similarities and differences in sexology education across world regions, as well as more in depth, qualitative analyses of the contextual and cultural impacts on sexology education within each region. The contributors to this special issue describe strengths and areas for growth from a global perspective and in more depth in each region. The special issue reinforces the link between the importance of professional training in clinical sexology and improving sexual health at a population level.

Advocacy with policymakers, including politicians and health administrators, is a necessary next step to make significant changes to the integration of clinical sexology education across health professions. This advocacy would start with educating policymakers about the links between sexual health and public health and helping them to increase their openness to centering sexual health as a major public health concern. In the few locations worldwide where sexology is a formally recognized profession (e.g. Quebec), it requires legislation. This may seem a daunting task, but even in cultures and religions that are perceived or interpreted as sex negative, there are proponents of sexual health.

Advancing the field of sexology is vital to the improvement of sexual health and to the well-being of all individuals, in line with the WAS mission. This special issue provides foundational knowledge about clinical sexology training across all world regions. With this foundation, we can start the work of developing international guidelines for professional education and accreditation of clinical sexology programs. With this in place, the field will be better situated to approach policymakers and advocate for the recognition of sexology as a profession, as well as the integration of sexual health education across health professions. We hope readers find the information provided in this special issue inspiring, informative, and encouraging, and that readers will take up the call to advance professional education in clinical sexology in their respective regions.

## Notes

1 GLObal Professional Education in Sexology.

2 https://www.worldsexualhealth.net/declarations.
